# Analysis of diverse factors influencing the health status as well as medical and health service utilization in the floating elderly of China

**DOI:** 10.1186/s12913-021-06410-7

**Published:** 2021-05-08

**Authors:** Yuwei Fu, Weiwei Lin, Yuan Yang, Ranran Du, Dongping Gao

**Affiliations:** Chinese Academy of Medical Sciences-Peking Union Medical College, Institute of Medical Information, 3 Yabao Rd, Beijing, 100020 People’s Republic of China

**Keywords:** Floating elderly, Self-rated health, Chronic disease, Physical examination, Medical and health service utilization

## Abstract

**Background:**

Based on the “China Migrants Dynamic Survey-Special investigation on Floating Elderly in 8 megacities in 2015”, the health status and the utilization of medical and health services in floating elderly were described and analyzed.

**Objective:**

Scientific basis and critical suggestions are provided for improving the utilization level of medical and health services in the floating elderly and designing targeted health policies to improve their well-being.

**Methods:**

The rank-sum test and Pearson χ^2^ test were used to compare the health status of floating elderly with different characteristics. Thereafter based on Andersen model, floating characteristics were added and binary logistic regression was used to explore the influencing factors of medical and health service utilization in the floating elderly.

**Results:**

About 94.7% of the floating elderly were self-assessed as healthy/basically healthy. About 24.2% had hypertension or diabetes as diagnosed by the qualified doctors. About 7% suffered from diseases that required hospitalization. Only 28.6% of the floating elderly with hypertension or diabetes had visited a doctor for follow-up. In the case of minor ailments, only 48.7% decided to visit the clinics. Approximately 70.7% of the floating elderly had used in-patient services when they suffered from diseases requiring hospitalization.

**Conclusion:**

The floating elderly were observed to be generally in good health but a high prevalence of hypertension or diabetes was observed among them. The cultivation of health awareness was found to be of great significance contributing to the improvement of the overall health level among the floating elderly. The basic medical insurance coverage was low, and the medical and health services were found to be severely underutilized. Adequate social support can promote the health of the floating elderly and improve their utilization of medical and health services. The floating reasons, scope and years of the elderly significantly affected their health status and the utilization rate of the basic public health services.

## Introduction

In China, citizens aged 60 and above are defined as the elderly (GB/T 24433–2009). The floating elderly refers to the population aged 60 or above who have lived in the local area for 1 month or more and are not registered in the district (county, city). With the rapid development of urbanization and aging population in China, the proportion of the elderly among the floating population has been increasing rapidly. According to the China Floating Population Development Report 2018, the number of the floating elderly in China has already entered a rapid growth stage in 2000 and has increased to 13.04 million in 2015, accounting for 5.3% of the national floating population, with an average annual growth rate of approximately 6.6% [1]. In 2018, the European Office of the World Health Organization (WHO) pointed out in the report “Health of older refugees and migrants”, that population aging and floating itself are a complex multi-dimensional process, and the needs of elderly refugees and migrants must be included in the scope of the whole European aging policy.

However, for the overall health status of the floating elderly, Yang JH (2018) declared that the health management of the floating elderly is poor, and less than half of them have established health records [2]. Song QC (2018) found that for the elderly, with increase in age, the physiological functions of organs may start degenerating, and the immunity can substantially decline, thereby leading to an increasing risk of various degenerative diseases such as diabetes [3]. Zhang JR (2017) found that because of rapid changes in the economic conditions, living environment and social roles, the floating elderly may face more hurdles in the utilization of medical and health services than the general elderly or other floating population, for example, the utilization rate of health services of the elderly floating population is low, and the demand for off-site medical treatment is difficult to meet [4]. Choi SH (2012) also stated that the floating elderly have their own particularities, including greater health risks, more unfavorable socio-economic status, language barriers and low health literacy, cultural factors affecting health seeking behavior, and social psychological vulnerability and discrimination [5]. Chu B (2015) found that the health awareness of the floating elderly is relatively weak, and the choice of medical institutions is irrational [6].

Kristiansen M (2016) found that the incidence and mortality rate of floating elderly in Europe were the same as those in the local population, but the relative importance, severity and age of onset were different. However, with the increase of age, the gap between the floating elderly and the local population was widening, and there were also great differences among the floating elderly themselves [7]. Gubernskaya Z (2015) also found that the older the migrants were, the greater the risk of poor health in their later years, which may be related to their long-term adverse conditions in the inflow area and limited opportunities for health care [8]. González HM (2011) also stated that the floating years, the age of migration and the floating reasons had a significant impact on the health and quality of life of the floating elderly. However, some studies had come to the opposite conclusion: the longer the migrants lived in the inflow area, the better the health status of them. This may result from that the more abundant social and economic resources and cultural adaptation played an important role in the later life of the migrants [9].

At present, there is no comprehensive study to investigate the influence of the elderly’s floating characteristics on their health status as well as utilization of medical and health services. It is rare to combine the two attributes of age and mobility. Based on this, this paper intends to select age and many related variables representing the floating pattern to comprehensively investigate the health status as well as utilization of medical and health services. This may aid to decipher the various influencing factors affecting their overall wellness. The findings provide a strong basis for improving the health level of the floating elderly and formulating novel policies related to their health status.

## Methods

### Data source

This study used the latest special survey data of floating elderly in China, “China Migrants Dynamic Survey-Special investigation on Floating Elderly in 8 megacities in 2015 [10]“, to analyze the health status and utilization of medical and health services among the floating elderly. The overall sample is all the floating population aged 60 or above in 8 megacities including Beijing, Shanghai, Dalian, Wuxi, Hangzhou, Hefei, Guangzhou and Guiyang. Stratified, multi-stage and population proportion sampling (PPS) was used in this survey. In the first stage, villages and towns / streets were selected; in the second stage, village committees / neighborhood committees were selected; in the third stage, individual respondents were selected. A total of 2279 people met the study requirements.

### Theoretical basis

**Fig. 1:**
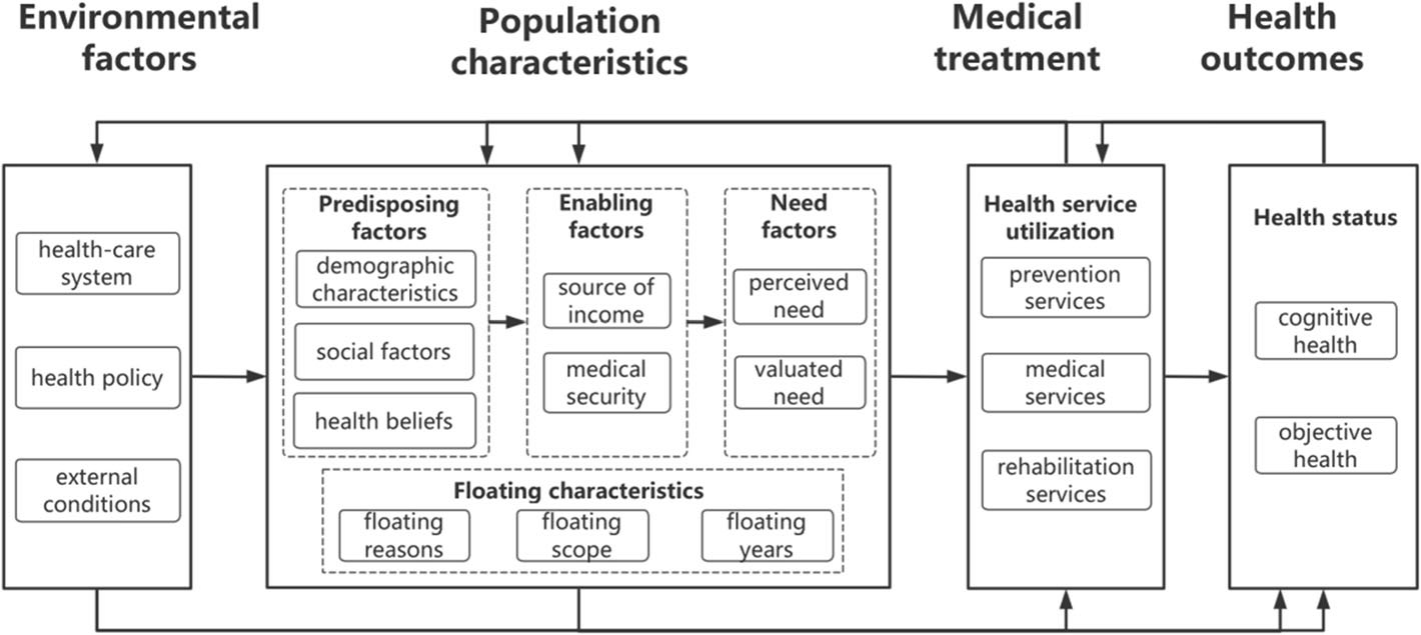
The framework of health service utilization model after adjustment

The Behavioral Model of Health Service Utilization (Andersen Model) was first proposed in 1968. Since its establishment, it has been widely used in medical and health services utilization research and health system evaluation [11]. According to the Anderson model, the utilization of personal medical and health services can be predominantly determined by three different factors: predisposing factors, enabling factors and need factors. Predisposing factors include demographic characteristics (age, gender, etc.), social factors (ethnic groups, education, occupation, marital status, social interaction, etc.) and health beliefs (attitudes, values, and knowledge of health systems, etc.). The enabling factors include personal/family resources (household income, health insurance, etc.) and community resources (access to health service, such as distance to the nearby hospital, waiting time to see a doctor, etc.). Finally, the need factors include cognition and objective evaluation of medical and health services. Based on Andersen model, this study utilizes the particularity of the research object and the actual content of the survey data into consideration so as to adjust the model. Therefore, the different floating characteristics including floating reasons, floating scope and floating years were added to the model and the theoretical frame diagram was established, as shown in Fig. 1.

### Conceptual model

#### Measurements of health status of floating elderly

Health has the dual characteristics of subjectivity and objectivity [12]. The subjective health and objective health of the floating elderly have been evaluated from the perspectives of individuals and professionals, including self-assessed health parameters, hypertension or diabetes diagnosed by doctors and diseases requiring hospitalization within 1 year, so as to comprehensively measure the overall health status among the floating elderly. The classification codes have been shown in Table 1.

**Table 1:** Evaluation indicators and influencing factors of medical and health service utilization

Variable names	Variable description	Group standard
**Evaluation indicators**		
Physical examination	yes = 1, can’t remember/no = 0	custom
Follow-up for hypertension/diabetes	yes = 1, no = 0	survey file
Treatment for minor ailments	active treatment (see a doctor, buy medicine or self-medicate, bring medicine from hometown) =1, negative attitude (not treat, buy health supplements, others) =0	custom
Hospitalization within one year	yes = 1, no = 0	survey file
**Predisposing factors**		
Age	low-age elderly (60 ~ 69) =1, middle-age elderly (70 ~ 79) =2, high-age elderly (> = 80) =3	custom
Gender	male = 1, female = 2	GB/T 2261.1–2003
Ethnic groups	Han = 01, Mongol = 02, Hui = 03, Miao = 06, Yi =07, Zhuang = 08, Buyi = 09, Chaoxian = 10, Manchu = 11, Dong =12, Tujia =15, others = 99	GB/T 3304–1991
Education level	graduate = 1, undergraduate college = 2, specialized subject in college = 3, high school/technical secondary school = 4, junior high school = 7, primary school = 8, not been to school = 9	GB/T 4658–2006
Marital status	unmarried = 1, married (first marriage, remarriage, marriage after divorce) =2, widowed = 3, divorced = 4	GB/T 2261.2–2003
Number of friends there	none = 1, 1 ~ 5 = 2, 6 ~ 10 = 3, > 10 = 4	custom
Household registration type	non-agricultural (non-agricultural, resident change from agricultural/non-agricultural) =1, agricultural = 0	custom
Average exercise time daily	0 ~ 30mins = 1, 31 ~ 60 mins = 2, > 60 mins = 3	custom
Health knowledge acquisition	1 point for each type of knowledge, group by total score, 0 point = 1, 1 ~ 4 point = 2, 5 ~ 9 point = 3	custom
**Enabling factors**		
Main economic source	labor income = 1, savings = 2, pension = 3, subsistence allowance = 4, rent = 5, family members, others = 9	survey file
Insurance type	new rural cooperation = 1, cooperation between urban and rural residents = 2, medical insurance for urban residents = 3, medical insurance for urban workers = 4, free medical service = 5, uninsured (unclear, none of these) =9	GB/T 31596.4–2015
Insurance location	native = 1, registered permanent residence = 2, others = 9	survey file
**Need factors**		
Self-rated health	healthy/basically healthy = 1, not healthy but able to take care of themselves = 2, cannot take care of themselves = 3	GB/T 2261.3–2003
Hypertension or diabetes	yes = 1, no = 0	survey file
Diseases requiring hospitalization	yes = 1, no = 0	survey file
**Floating characteristics**		
Floating reasons	work and do business = 1, take care of children/grandchildren or float with family members =2, treat diseases = 3, live a retired life = 4, others = 9	custom
Floating scope	trans-provincial = 1, trans-city = 2, trans-county = 3	survey file
Floating years	< 1 year = 1, > = 1& < 5 years = 2, > = 5& < 10 years = 3, > = 10 years = 4	custom

#### Evaluation indicators of the medical and health service utilization

Medical and health service utilization mainly refers to the utilization of prevention, medical treatment and rehabilitation services for the management of chronic diseases [13]. It has been well established that with the increase of age, the body functions gradually decrease, and the adaptability of the elderly population to the external environment can deteriorate significantly [14]. Hence the elderly population become more susceptible to several diseases and easily prone to minor ailments, and develop an increased risk of acquiring major diseases and common chronic diseases. However, physical examination can effectively identify high-risk groups, which may be significantly beneficial for disease prevention [15]. In fact, the early hospitalization for diseases or injuries, treatment for minor ailments and follow-up for hypertension, diabetes or other life-style related diseases reflect the optimum utilization of medical and rehabilitation services by the floating elderly. As a result, free physical examination, follow-up for hypertension or diabetes, treatment for minor ailments and hospitalization within 1 year were selected to carefully evaluate the utilization of medical and health services by the floating elderly in this study.

#### Influencing factors of medical and health service utilization

According to the adjusted Anderson model, age, gender, ethnic groups, education level and marital status were selected among the predisposing factors. The “number of friends there” has been used to reflect the social interaction among the floating elderly. In addition, considering the imbalanced infrastructure development between urban and rural areas in China, the household registration type was also incorporated into the model. The average exercise time daily and health knowledge acquisition were used to understand the health beliefs among the subjects. Health knowledge in the investigation includes nine kinds, such as occupational disease prevention, nutritional health knowledge, reproduction and contraception/prenatal and postnatal care, chronic disease prevention and control, smoking control, mental disorders prevention and control, tuberculosis prevention and control, sexually transmitted disease (STD) or acquired immune deficiency syndrome (AIDS), prevention and control of other infectious diseases. The main economic source, insurance type and insurance location were also used to describe the various enabling factors. However, in terms of the need factors, the health status of the floating elderly may also exhibit a significant great impact on their medical and health service utilization capability. Therefore, self-assessed health, hypertension or diabetes diagnosed by doctors and diseases requiring hospitalization within 1 year were also selected as the independent variables. In addition, the floating characteristics including floating reasons, floating scope and floating years were used for analysis. The evaluation indicators and influencing factors of medical and health service utilization have been detailed in Table 1.

### Statistical methods

A general descriptive analysis was applied to analyze the basic situations and health status of the floating elderly. The rate and composition ratio were used for the enumeration data. The health status of the floating elderly with different characteristics was determined by rank-sum test and Pearson χ^2^ test. When the frequency is less than 5, Fisher’s exact test was used for analysis. A univariate logistic regression was applied to identify the potential influencing factors affecting medical and health service utilization, and binary logistic regression was used for model fit studies. *P* < 0.05 indicated that the difference was statistically significant. All statistical analyses were performed in Statistical Product and Service Solutions (SPSS) version 22.0.

## Results

### Basic characteristics of the floating elderly

In this study, people aged 60 ~ 69 were defined as “low-age elderly”, who aged 70 ~ 79 were called “middle-age elderly”, and people aged 80 or above were defined as “high-age elderly”. The health status of 2279 floating elderly with age ranging from 60 to 96 years old was analyzed, among which the low-age elderly accounted for the highest proportion (about 77.4%). The number of men and women is roughly equal. The education level was mainly limited to primary and junior high school, accounting for 27.8 and 28.9%, respectively. About 84.9% of the floating elderly were married. In terms of the social interaction, about 12.3% of the floating elderly had no friends there, and 41.2% of the floating elderly had around 1 ~ 5 friends. In addition, 26.3% of the floating elderly actively participated in exercise for 30 min or less every day, and 39.5% took exercise for 31 to 60 min on an average. The health knowledge acquisition of floating elderly was generally observed to be poor. Among the nine types of health knowledge, about 60.9% of floating elderly were found to be exposed to only 4 types or even less. The main source of the income of the floating elderly subjects was pension, accounting for 53.6, and 23.9% of the floating elderly mainly depended on family members to meet their financial needs.

The coverage of basic medical insurance for the floating elderly was relatively low, and about 21.5% of the floating elderly were not covered by any type of medical insurance. A very substantial proportion of the floating elderly reported having a good health status. Approximately, 94.7% of the floating elderly were self-assessed as healthy/basically healthy, and only 0.8% of the floating elderly could not take proper care of themselves. However, about 24.2% of the floating elderly had hypertension or diabetes as diagnosed by the qualified doctors. Moreover, about 7% of the floating elderly suffered from diseases that required hospitalization. The major reasons for migration of the elderly subjects were mainly related to taking care of their children/grandchildren or floating along with their family members, accounting for 45.6%. About 28.9% of the elderly floated for living a better retired life. The floating scope was predominantly found to be trans-provincial floating, thus accounting for about 82.6%. The floating time of the elderly was relatively longer on the whole, for instance 87.2% of them have been floating for more than 1 year, and 53.8% of them have been floating for greater than 5 years. The basic characteristics of the floating elderly is displayed in Table 2.

**Table 2:** The basic characteristics of the floating elderly

Variable	Value	N (%)
Education level		
	undergraduate college and above	326 (14.3)
	specialized subject in college	34 (1.5)
	high school / technical secondary school	1221 (53.6)
	junior high school	659 (28.9)
	primary school	633 (27.8)
	not been to school	310 (13.6)
Marital status		
	married	1935 (84.9)
	widowed	307 (13.5)
	divorced	34 (1.5)
Number of friends there		
	none	278 (12.3)
	1 ~ 5	930 (41.2)
	6 ~ 10	603 (26.7)
	> 10	444 (19.7)
Household registration type		
	agricultural	1053 (46.2)
	non-agricultural	1226 (53.8)
Average exercise time daily		
	0 ~ 30 mins	600 (26.3)
	31 ~ 60 mins	900 (39.5)
	> 60 mins	775 (34)
Health knowledge acquisition		
	0 point	91 (11.1)
	1 ~ 4 point	409 (49.8)
	5 ~ 9 point	321 (39.1)
Insurance type		
	new rural cooperation	796 (34.9)
	cooperation between urban and rural residents	80 (3.5)
	medical insurance for urban residents	235 (10.3)
	medical insurance for urban workers	617 (27.1)
	free medical service	59 (2.6)
	uninsured	489 (21.5)
Main economic income		
	labor income	326 (14.3)
	savings / rent	34 (1.5)
	pension	1221 (53.6)
	subsistence allowance	34 (1.5)
	family members	545 (23.9)
Insurance location		
	native	58 (2.5)
	registered permanent residence	1723 (75.6)
Self-rated health		
	healthy / basically healthy	2158 (94.7)
	not healthy but able to take care of themselves	100 (4.4)
	cannot take care of themselves	18 (0.8)
Hypertension or diabetes		
	yes	552 (24.2)
	no	1727 (75.8)
Diseases requiring hospitalization		
	yes	159 (7)
	no	2120 (93)
Floating reasons		
	work and do business	395 (17.3)
	take care of children/grandchildren or float with family members	1040 (45.6)
	treat diseases	15 (0.7)
	live a retired life	658 (28.9)
Floating scope		
	trans-provincial	1882 (82.6)
	trans-city	287 (12.6)
	trans-county	110 (4.8)
Floating years		
	< 1 year	291 (12.8)
	> = 1& < 5 years	762 (33.4)
	> = 5& < 10 years	628 (27.6)
	> = 10 years	598 (26.2)

### Analysis on the health status of the floating elderly with different characteristics

#### Self-rated health

A very substantial proportion of the floating elderly reported having a good health status, with the proportion of healthy/basically healthy above 89%. The rank-sum test was conducted for self-rated health and each factor was taken into consideration respectively. The results clearly showed that there were significant differences (*P* < 0.05). The self-assessed health status of the floating elderly with different characteristics has been shown in Table 3.

**Table 3:** Self-rated health status of floating elderly with different characteristics n (%)

Variable	Value	Healthy/basically healthy	Not healthy but able to take care of themselves	Cannot take care of themselves	Test statistics	*P*
Age					66.947	< 0.001
	low-age elderly	1704 (79.0)	50 (50.0)	6 (33.3)		
	middle-age elderly	392 (18.2)	41 (41.0)	9 (50.0)		
	high-age elderly	62 (2.9)	9 (9.0)	3 (16.7)		
Education level					20.438	0.002
	undergraduate college and above	119 (5.5)	4 (4.0)	0 (0.0)		
	specialized subject in college	160 (7.4)	2 (2.0)	0 (0.0)		
	high / technical secondary school	377 (17.5)	10 (10.0)	5 (27.8)		
	junior high school	625 (29.0)	30 (30.0)	4 (22.2)		
	primary school	597 (27.7)	30 (30.0)	3 (16.7)		
	not been to school	280 (13.0)	24 (24.0)	6 (33.3)		
Household registration type					5.707	0.017
	agricultural	985 (45.6)	54 (54.0)	13 (72.2)		
	non-agricultural	1173 (54.4)	46 (46.0)	5 (27.8)		
Marital status					24.474	< 0.001
	married	1850 (85.7)	74 (74.0)	8 (44.4)		
	widowed	274 (12.7)	24 (24.0)	9 (50.0)		
	divorced	31 (1.4)	2 (2.0)	1 (5.6)		
Main economic source					35.844	< 0.001
	labor income	320 (14.8)	6 (6.0)	0 (0.0)		
	savings / rent	33 (1.5)	1 (1.0)	0 (0.0)		
	pension	1173 (54.4)	43 (43.0)	5 (27.8)		
	subsistence allowance	29 (1.3)	5 (5.0)	0 (0.0)		
	family members	496 (23.0)	39 (39.0)	10 (55.6)		
Number of friends there					24.114	< 0.001
	none	250 (11.7)	19 (19.6)	9 (50.0)		
	1 ~ 5	876 (40.9)	50 (51.5)	4 (22.2)		
	6 ~ 10	582 (27.2)	17 (17.5)	4 (22.2)		
	> 10	432 (20.2)	11 (11.3)	1 (5.6)		
Average exercise time daily					49.607	< 0.001
	0 ~ 30 mins	537 (24.9)	48 (48.0)	15 (83.3)		
	31 ~ 60 mins	864 (40.1)	33 (33.0)	3 (16.7)		
	> 60 mins	756 (35.0)	19 (19.0)	0 (0.0)		
Floating reasons					157.743	< 0.001
	work and do business	383 (17.7)	11 (11.0)	1 (5.6)		
	take care of children/ grandchildren or float with family members	1018 (47.2)	21 (21.0)	0 (0.0)		
	treat diseases	6 (0.3)	3 (3.0)	6 (33.3)		
	live a retired life	590 (27.3)	57 (57.0)	11 (61.1)		
Floating scope					9.598	0.008
	trans-provincial	1792 (83.0)	73 (73.0)	14 (77.8)		
	trans-city	268 (12.4)	16 (16.0)	3 (16.7)		
	trans-county	98 (4.5)	11 (11.0)	1 (5.6)		
Floating years					9.556	0.023
	< 1 year	281 (13.0)	7 (7.0)	3 (16.7)		
	> = 1& < 5 year	709 (32.9)	45 (45.0)	8 (44.4)		
	> = 5& < 10 year	604 (28.0)	19 (19.0)	4 (22.2)		
	> = 10 year	564 (26.1)	29 (29.0)	3 (16.7)		

The results of paired comparison between the classes indicated that the self-assessed health status of the low-age floating elderly was significantly better as compared to that of the middle-age and high-age floating elderly, and there was no significant difference between the middle-age and high-age subjects. Moreover, in comparison with the floating elderly with specialized education in college, high school or technical secondary school education, the floating elderly who had not been to school rated their health status as relatively poor. The self-assessed health status of the floating elderly with non-agricultural household registration was relatively better than that of agricultural household registration. Furthermore, as compared with the married floating elderly, the widowed floating elderly had a poor self-assessed health status. The floating elderly whose main economic source was labor income had a better self-assessed health status than those who were dependent on the subsistence allowance or the family members for financial support. The floating elderly living on pension were also observed to be in a better health than the floating elderly living on the support of family members. In terms of social interaction, the self-assessed health status of the floating elderly with one or more friends was found to be significantly better than those of the subjects without any friends. Furthermore, as compared with the floating elderly who did not carry out any regular exercise every day or whose average exercise time was less than 30 mins, the floating elderly who performed regular exercise for more than 30 mins had a significantly better self-rated health status. Interestingly, in terms of the floating characteristics, the self-rated health status of the elderly who relocated for treatment purposes or living a retired life was far worse than those subjects who took care of their children/grandchildren or floated with their family members. In addition, for the floating elderly who carried out trans-provincial floating, the self-assessed health status was significantly better than those who executed trans-county floating. The floating elderly who have been continuously floating for 5 years or more but less than 10 years were observed to display a significantly better self-assessed health status as compared to those who have been on the floating for 1 year or more but less than 5 years.

#### Hypertension or diabetes

**Fig. 2:**
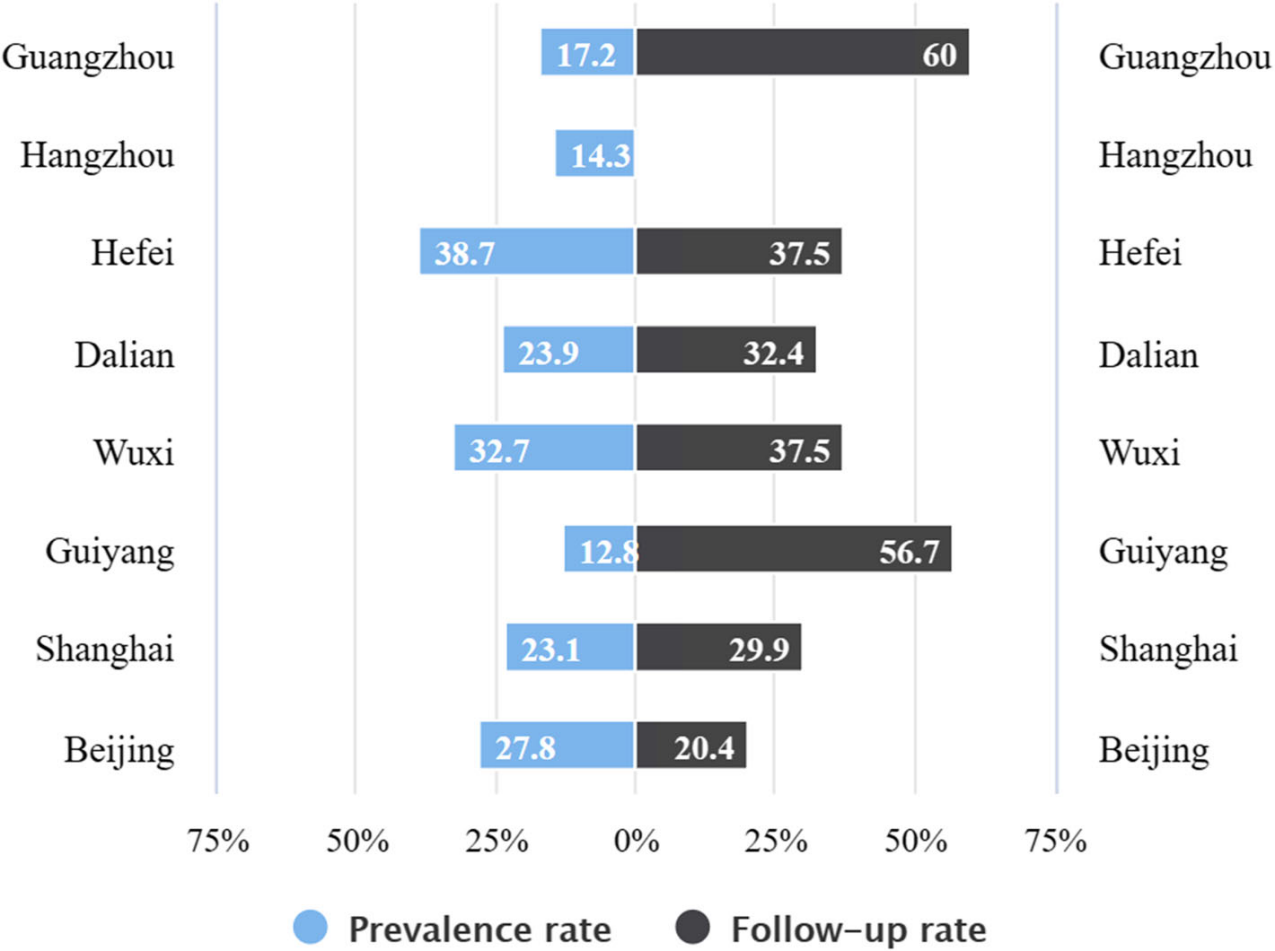
Prevalence and follow-up rate of hypertension or diabetes among the floating elderly in different megacities

The three megacities with the highest prevalence of hypertension or diabetes among the floating elderly were Hefei, Wuxi, and Beijing, respectively. The prevalence in Guiyang was the lowest, about 12.8%. The prevalence and follow-up rate of hypertension or diabetes among the floating elderly in different megacities has been shown in Fig. 2. All the data was analyzed using Pearson χ^2^ test or Fisher’s exact test. There were significant differences observed in the prevalence of hypertension or diabetes (*P* < 0.05). The prevalence of hypertension or diabetes in the floating elderly with different characteristics has been shown in Table 4.

**Table 4:** Hypertension or diabetes prevalence in the floating elderly with different characteristics n (%)

Variable	Value	Hypertension or diabetes		χ^2^	*P*
		yes	no		
Age				27.672	< 0.001
	low-age elderly	382 (69.2)	1378 (79.9)		
	middle-age elderly	144 (26.1)	298 (17.3)		
	high-age elderly	26 (4.7)	48 (2.8)		
Education level				17.079	0.007
	undergraduate college and above	44 (8.0)	79 (4.6)		
	specialized subject in college	39 (7.1)	123 (7.1)		
	high school/technical secondary school	106 (19.2)	286 (16.6)		
	junior high school	164 (29.7)	495 (28.7)		
	primary school	129 (23.4)	501 (29.1)		
	not been to school	70 (12.7)	240 (13.9)		
Household registration type				34.802	< 0.001
	agricultural	195 (35.3)	857 (49.7)		
	non-agricultural	357 (64.7)	867 (50.3)		
Main economic source				73.425	< 0.001
	labor income	37 (6.7)	289 (16.8)		
	savings / rent	5 (0.9)	29 (1.7)		
	pension	370 (67.0)	851 (49.4)		
	subsistence allowance	11 (2.0)	23 (1.3)		
	family members	115 (20.8)	430 (24.9)		
Insurance type				42.233	< 0.001
	new rural cooperation	157 (28.4)	639 (37.1)		
	cooperation between urban and rural residents	22 (4.0)	58 (3.4)		
	medical insurance for urban residents	55 (10.0)	180 (10.4)		
	medical insurance for urban workers	198 (35.9)	419 (24.3)		
	free medical service	23 (4.2)	36 (2.1)		
	uninsured	97 (17.6)	392 (22.7)		
Floating reasons				25.291	< 0.001
	work and do business	59 (10.7)	336 (19.5)		
	take care of children/grandchildren or float with family members	269 (48.7)	770 (44.7)		
	treat diseases	4 (0.7)	11 (0.6)		
	live a retired life	171 (31.0)	487 (28.2)		
Floating years				9.088	0.028
	< 1 year	58 (10.5)	233 (13.5)		
	> = 1& < 5 year	181 (32.8)	581 (33.7)		
	> = 5& < 10 year	144 (26.1)	483 (28.0)		
	> = 10 year	169 (30.6)	427 (24.8)		

The Pairwise comparison results clearly suggested that the prevalence rate of hypertension or diabetes in the middle-age and high-age floating elderly was significantly higher than that in the low-age floating elderly. Furthermore, upon comparison with the floating elderly with primary school education, the floating elderly with a bachelor’s degree had a significantly higher prevalence rate of hypertension or diabetes. The proportion of hypertension or diabetes among the elderly with non-agricultural household was found to be higher than that of the elderly with agricultural household. In addition, compared with the floating elderly whose main economic source was labor income, those who were dependent on family members, pension and subsistence allowance displayed a higher rate of hypertension or diabetes. The prevalence of hypertension or diabetes in the pension group was significantly higher than those who were economically dependent on the family members. The floating elderly who actively participated in free medical service or medical insurance for urban workers had a higher prevalence of hypertension or diabetes than those who participated in the new rural cooperative insurance or belonged to the non-insured group. Additionally, in terms of floating characteristics, upon comparison with the elderly floating for working and business, the elderly who migrated to take care of their children/grandchildren or floated on a regular basis with family members displayed a significantly higher incidence of hypertension or diabetes. The proportion of hypertension or diabetes in the elderly who had been continuously floating for 10 years or more was observed to be significantly higher than those who had been floating for less than 1 year. The chi-square trend test results also suggested that the prevalence rate of hypertension or diabetes among the floating elderly clearly showed an increasing trend with the increase of age, education level and the number of floating years.

#### The prevalence of diseases requiring hospitalization within one year

**Fig. 3:**
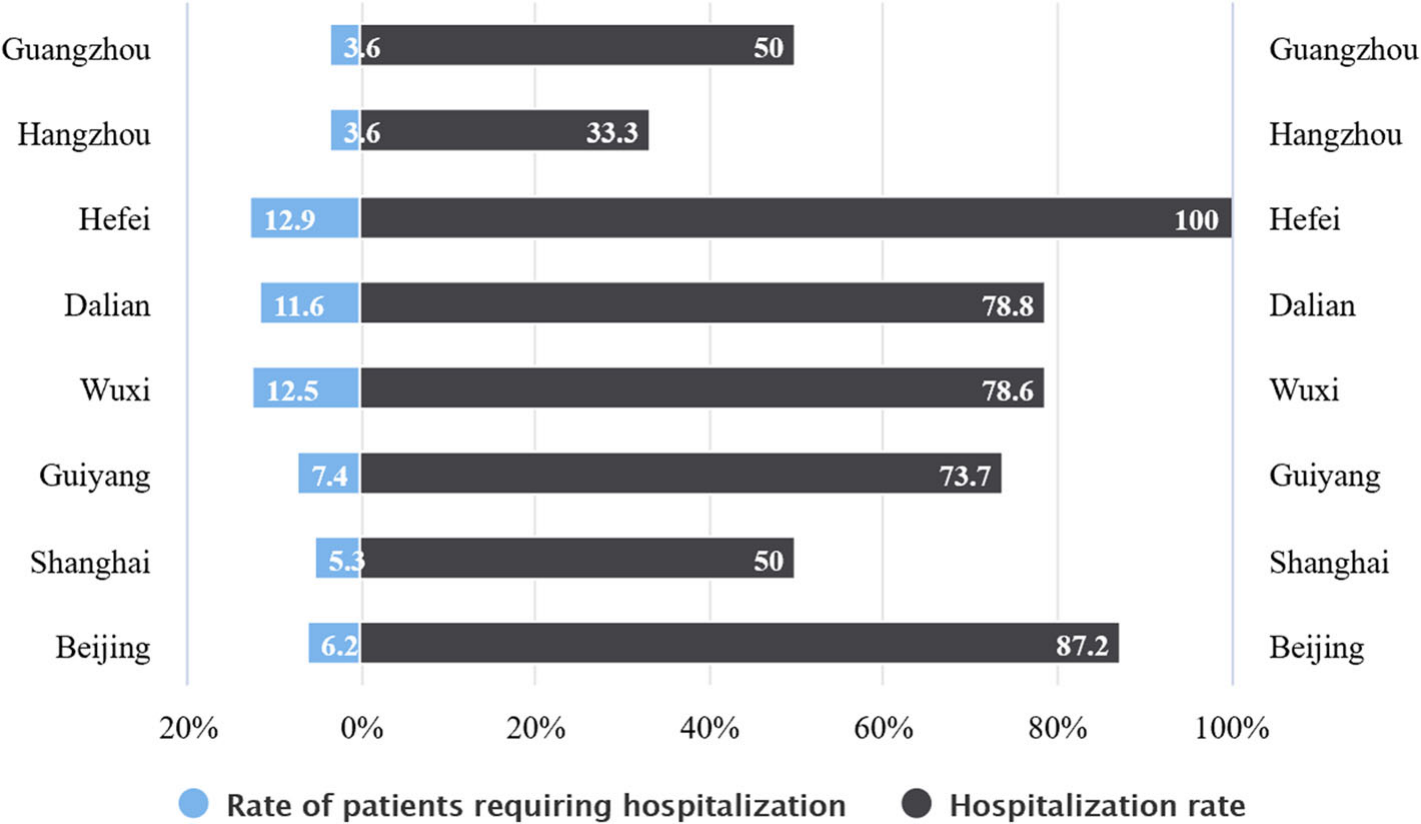
The rate of patients requiring hospitalization and the hospitalization rate of the floating elderly in different megacities within one year

The three megacities with the highest incidence of diseases which requiring hospitalization among the floating elderly within 1 year were Hefei, Wuxi, and Dalian, all of which were noted to be above 10%. For instance, Guangzhou and Hangzhou had the lowest rate of the patients requiring hospitalization within 1 year for the floating elderly, at only 3.6%. The rate of the patients requiring hospitalization and the hospitalization rate of the floating elderly in the different megacities within 1 year has been displayed in Fig. 3. Moreover, Pearson χ^2^ test or Fisher exact test results clearly showed that there were statistically significant differences (*P* < 0.05). The rate of diseases requiring hospitalization among the floating elderly population with different characteristics within 1 year has been shown in Table 5.

**Table 5:** The status of diseases requiring hospitalization among the floating elderly with different characteristics within one year n (%)

Variable	Value	Diseases requiring hospitalization		χ^2^	*P*
		yes	no		
Age				37.139	< 0.001
	low-age elderly	95 (59.7)	1634 (78.4)		
	middle-age elderly	49 (30.8)	391 (18.8)		
	high-age elderly	15 (9.4)	59 (2.8)		
Main economic source				16.65	0.009
	labor income	9 (5.7)	303 (14.5)		
	savings / rent	2 (1.3)	32 (1.5)		
	pension	92 (57.9)	1115 (53.5)		
	subsistence allowance	6 (3.8)	28 (1.3)		
	family members	40 (25.2)	502 (24.1)		
Number of friends there				12.077	0.007
	none	33 (20.9)	244 (11.8)		
	1 ~ 5	64 (40.5)	853 (41.3)		
	6 ~ 10	37 (23.4)	554 (26.8)		
	> 10	24 (15.2)	413 (20.0)		
Insurance type				13.119	0.02
	new rural cooperation	48 (30.2)	736 (35.3)		
	cooperation between urban and rural residents	6 (3.8)	73 (3.5)		
	medical insurance for urban residents	10 (6.3)	222 (10.7)		
	medical insurance for urban workers	61 (38.4)	551 (26.4)		
	free medical service	6 (3.8)	53 (2.5)		
	uninsured	28 (17.6)	449 (21.5)		
Floating reasons				76.982	< 0.001
	work and do business	13 (8.2)	368 (17.7)		
	take care of children/grandchildren or float with family members	52 (32.7)	974 (46.7)		
	treat diseases	10 (6.3)	5 (0.2)		
	live a retired life	79 (49.7)	576 (27.6)		
Floating scope				8.363	0.015
	trans-provincial	128 (80.5)	1722 (82.6)		
	trans-city	16 (10.1)	268 (12.9)		
	trans-county	15 (9.4)	94 (4.5)		

The pairwise comparison results suggested that the rate of diseases requiring hospitalization within 1 year among the middle-age and high-age floating elderly was significantly higher than that of the low-age elderly. In addition, upon comparison with the floating elderly with 6 or more active friends, the rate of diseases requiring hospitalization within 1 year was lower as compared to that of the floating elderly without any friends. Chi-square trend test results also indicated that the rate of diseases requiring hospitalization within 1 year can significantly increase with the increase of age or the decrease in number of the friends. Moreover, when compared with the floating elderly whose main economic source was the subsistence allowance, the rate of diseases requiring hospitalization within 1 year was found to be significantly higher than those subjects who primarily relied on their labor income. The rate of diseases requiring hospitalization within 1 year among the floating elderly with different insurance types was noted to be generally different, but the pairwise comparison clearly showed that the difference was not statistically significant. Interestingly, in the terms of floating characteristics, the rate of diseases requiring hospitalization within 1 year was found to be significantly higher than those elderly subjects who primarily migrated for treating illness or living a retired life. Among them, the rate of diseases requiring hospitalization within 1 year was observed to be highest for the elderly subjects who decided to float for medical treatment. Finally, compared with the floating elderly who carried out trans-provincial or trans-city floating, the rate of diseases requiring hospitalization within 1 year was lower than those elderly who carried out trans-country floating.

### The utilization status of medical and health service for the floating elderly

It was found that only 659 of the 2279 floating elderly had participated in the free physical examination organized by community health service stations or centers, and the free physical examination coverage rate was observed only to be 29%. Interestingly, among the 552 floating elderly with hypertension or diabetes in this study, only 158 of them had visited a doctor for follow-up, thus accounting for 28.6%. In the case of minor ailments, 97.6% of the floating elderly sought some form of treatment. Among them, 48.7% decided to visit the clinics, 47.3% bought drugs or self-medicated themselves, and 1.6% opted to receive drugs from their hometown. Overall, approximately 70.7% of floating elderly had used in-patient services for diseases requiring hospitalization. The detailed results are shown in Table 6.

**Table 6:** The utilization status of medical and health service for the floating elderly

Variable	Value	Number/n	Ratio /%
Free physical examination			
	yes	659	29
	no	1617	71
Follow-up for hypertension or diabetes			
	yes	158	28.6
	no	394	71.4
Treatment for minor ailments			
	visit the clinics	1108	48.7
	buy medicine / self-medicated	1077	47.3
	bring medicine from hometown	37	1.6
	buy health care products	3	0.1
	no treatment	33	1.4
	others	18	0.8
Hospitalization within one year			
	yes	133	70.7
	no	55	29.3

**Fig. 4:**
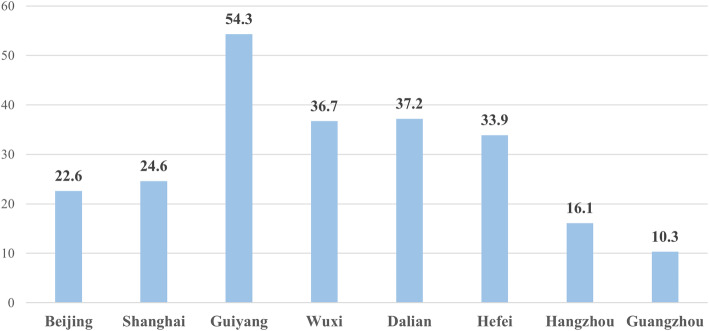
Participation rate of health examination of among the floating elderly in different megacities (%)

As displayed in Fig. 4, the physical examination coverage rate of the floating elderly in Guiyang was 54.3%, which was significantly higher than that reported in other megacities. In addition, Wuxi, Dalian, and Hefei were the next three megacities in ranking order, and the participation rates of physical examination for the floating elderly were found to be above 30%. The physical examination rate for the floating elderly in Guangzhou was the lowest, at only 10.3%.

The proportion of the floating elderly who had hypertension or diabetes diagnosed by doctors in Guangzhou and Guiyang using follow-up service was observed to be 60 and 56.7% respectively, which was significantly higher than noted in the other megacities. The follow-up rate for hypertension and diabetes among the floating elderly in Beijing was the lowest, at only 20.4%. The detailed data has been shown in Fig. 2. The proportion of the floating elderly in 8 megacities who opted to seek medical treatment actively when they were diagnosed with minor illnesses was over 96%, and there was no significant difference noted among the different megacities. As revealed in Fig. 3, the utilization rate of in-patient services for the floating elderly in Hefei and Beijing was obviously higher than that in other megacities when the subjects were suffering from diseases requiring hospitalization. The in-patient service utilization rate of the floating elderly in Hangzhou was found to be the lowest, only at 33.3%.

### Analysis of factors directly affecting medical and health service utilization for the floating elderly

#### Independent variables selection

After taking four indicators introduced in section 2.3.2 as dependent variables, univariate logistic regression analysis was carried out on the independent variables related to the various predisposing factors, enabling factors, need factors and floating characteristics so as to screen out the possible influencing factors. The number of graduate subjects was observed to be relatively small, so the group was combined with the undergraduate students and termed as undergraduate students and above. Among the main economic source, rent and savings were merged together and termed as savings/rent. There was a total of 12 independent variables with statistically significant difference observed during the physical examination of the floating elderly, including age group, number of active friends, household registration type, education level, health knowledge acquisition, daily average exercise duration, major income source, insurance type, insurance location, floating reasons, floating scope as well floating years. In addition, the factors that may affect the follow-up for hypertension or diabetes in the floating elderly were age group, number of friends, daily average exercise time and the reasons contributing to floating. The possible influencing factors for the treatment of the minor ailments included the number of active friends, major income source, insurance type, floating reasons, and hypertension or diabetes. Finally, the factors that may affect the hospitalization within 1 year in the floating elderly subjects were noted to be daily average exercise duration, major income source, insurance type as well as location, floating reasons and hypertension or diabetes.

### Logistic regression analysis

A binary logistic regression model was constructed separately for physical examination, follow-up for hypertension or diabetes, treatment for minor ailments and hospitalization within 1 year and their possible influencing factors, which were significant in univariate logistic regression analysis. The forward stepwise regression method that was based on the maximum likelihood estimation was selected. The test results indicated that the four models were statistically significant (*P* < =0.001), and met the best fit criteria (*P* > 0.05), with the prediction accuracy 69.8, 71.4, 97.6 and 70.7%, respectively. There was no multicollinearity noted among the different analyzed factors. The specific regression results of the model have been shown in Table 7.

**Table 7:** Binary logistic regression analysis on different influencing factors related to the medical and health services utilization among the floating elderly subjects

Model	Independent variable	*B*	*Wald χ*^*2*^	*P*	*OR (95%CI)*
Model 1	Number of friends there (*none* is as control)				
	1 ~ 5	0.368	0.859	0.354	1.445 (0.663,3.149)
	6 ~ 10	0.896	5.014	0.025	2.450 (1.118,5.368)
	> 10	0.724	3.057	0.080	2.063 (0.916,4.647)
	Health knowledge acquisition (*0 point* is as control)				
	1 ~ 4 point	1.695	9.742	0.002	5.447 (1.879,15.793)
	5 ~ 9 point	2.013	13.439	0.000	7.487 (2.552,21.968)
	Insurance location (*native* is as control)				
	registered permanent residence	1.027	5.361	0.021	2.792 (1.171,6.659)
	Floating scope (*trans-provincial* is as control)				
	trans-city	1.009	16.370	0.000	2.743 (1.682,4.472)
	trans-county	0.617	2.414	0.120	1.854 (0.851,4.040)
	Floating reasons (*work and do business* is as control)				
	take care of children/grandchildren or float with family members	0.590	4.723	0.030	1.804 (1.060,3.071)
	treat diseases	21.965	0.000	1.000	
	live a retired life	0.878	11.406	0.001	2.407 (1.446,4.006)
	Floating years (*< 1 year* is as control)				
	> = 1& < 5 year	0.181	0.257	0.612	1.198 (0.596,2.410)
	> = 5& < 10 year	0.727	4.139	0.042	2.069 (1.027,4.169)
	> = 10 year	0.661	3.379	0.066	1.937 (0.957,3.919)
	constant	−4.334	34.687	0.000	
Model 2	Number of friends there (*> 10* is as control)				
	none	−0.494	2.201	0.138	0.610 (0.317,1.172)
	1 ~ 5	−0.691	6.700	0.010	0.501 (0.297,0.845)
	6 ~ 10	−0.065	0.053	0.818	0.937 (0.539,1.629)
	Average exercise time daily (*> 60 mins* is as control)				
	0 ~ 30 mins	0.563	4.328	0.037	1.755 (1.033,2.983)
	31 ~ 60 mins	0.867	13.732	0.000	2.379 (1.504,3.762)
	constant	−1.070	18.684	0.000	
Model 3	Number of friends there (*none* is as control)				
	1 ~ 5	1.005	5.606	0.018	2.733 (1.189,6.282)
	6 ~ 10	0.106	0.072	0.789	1.112 (0.511,2.420)
	> 10	1.134	4.543	0.033	3.109 (1.096,8.821)
	Main economic source (*labor income* is as control)				
	savings / rent	18.239	0.000	0.998	
	pension	1.292	6.248	0.012	3.640 (1.322,10.025)
	subsistence allowance	0.741	0.441	0.507	2.098 (0.236,18.686)
	family members	1.597	6.990	0.008	4.938 (1.512,16.131)
	Floating reasons (*others* is as control)				
	work and do business	1.219	4.101	0.043	3.384 (1.040,11.013)
	take care of children/grandchildren or float with family members	1.003	5.515	0.019	2.727 (1.181,6.299)
	treat diseases	18.202	0.000	0.999	
	live a retired life	1.371	7.168	0.007	3.937 (1.444,10.738)
	constant	1.173	3.557	0.059	
Model 4	Main economic source (*labor income* is as control)				
	savings	−20.126	0.000	1.000	
	pension	1.836	6.005	0.014	6.274 (1.444,27.254)
	subsistence allowance	21.976	0.000	0.999	
	family members	2.257	7.478	0.006	9.557 (1.895,48.189)
	Hypertension or diabetes (*no* is as control)				
	yes	0.915	4.204	0.040	2.496 (1.041,5.983)
	constant	−1.077	2.473	0.116	

The factors that influenced whether the floating elderly could actively participate in the free physical examination include the number of active friends, health knowledge acquisition, insurance location, floating reasons, scope, and the number of years. The regression coefficients of the six factors were observed to be all positive. Among the predisposing factors, the probability of the floating elderly subjects with 6 ~ 10 or more friends attending the physical examination was 2.45 times and 2.06 times that of those subjects without any friends, respectively. In addition, compared to the floating elderly who had never acquired any substantial health related knowledge, the floating elderly with significant health knowledge score of 1 ~ 4 or 5 ~ 9 were 5.45 and 7.49 times more likely to choose to undertake physical examination, respectively. Moreover, in the terms of enabling factors, the floating elderly insured in registered permanent residence were significantly more likely to participate in free physical examination than those subjects who were insured in the local area. Furthermore, among the floating characteristics, the elderly who moved across megacities were 2.74 times more likely to participate in the physical examination than the elderly who primarily migrated only across the provinces. Interestingly, compared with the elderly who primarily floated for work or business, the elderly who floated to take care of their children/grandchildren or floated only with their family members were more likely to attend free physical examination. The probability of the elderly who have been floating around for 5 years or more and less than 10 years opting to actively participate in the health checkup was 2.07 times that of the elderly subjects who have been floating only for less than 1 year.

The number of friends there and average exercise time daily were observed as the main influencing factors determining the utilization of follow-up service for hypertension or diabetes among the floating elderly population. The probability of the floating elderly with 1 to 5 active friends availing the follow-up medical services for hypertension or diabetes was only 50.1% of those with more than 10 friends. The floating elderly who regularly exercised at least for 60 mins or less per day were more likely to use follow-up services for hypertension or diabetes than those subjects who exercised for more than 60 mins.

The factors affecting the treatment for minor illness of the floating elderly subjects included the number of active friends, the major income source, and floating reasons. Among the various predisposing factors analyzed, the probability of active treatment for minor ailments among the floating elderly with 1 ~ 5 or more than 10 friends in the area was found to be 2.73 times and 3.11 times of those without any friends, respectively. However, in terms of enabling factors, upon comparison with the floating elderly whose main income source was only labor income, the floating elderly relying on pension or financial support from the family members were more likely to seek medical advice and treatment based on their own initiatives, with a probability of 3.64 times and 4.94 times, respectively. Furthermore, upon consideration of various floating characteristics, the elderly who floated to work and to do business, or live a retired life and take care of their children/grandchildren or float with their family members were found to be significantly more inclined to choose active treatment when they were diagnosed with minor illness. In addition, in order to explore the differences in the treatment of minor diseases between the floating elderly with different characteristics, such as seeing a doctor, buying medicine or self-treatment, chi square test was conducted, and the results showed that there were significant differences (*P* < 0.05). The detailed results are shown in Table 8.

**Table 8:** Treatment for minor ailments of floating elderly with different characteristics n (%)

Variable	Value	Treatment for minor ailments				χ^2^ (P)
		see a doctor	buy medicine or self-medicate	bring medicine from hometown	not treat	
Education level						45.768 (< 0.001)
	undergraduate college and above	66 (6.0)	50 (4.6)	4 (10.8)	0 (0.0)	
	specialized subject in college	88 (7.9)	65 (6.0)	7 (18.9)	1 (3.0)	
	high school/technical secondary school	214 (19.3)	168 (15.6)	4 (10.8)	3 (9.1)	
	junior high school	313 (28.2)	323 (30.0)	11 (29.7)	6 (18.2)	
	primary school	269 (24.3)	330 (30.6)	9 (24.3)	15 (45.5)	
	not been to school	158 (14.3)	141 (13.1)	2 (5.4)	8 (24.2)	
Household registration type						19.719 (< 0.001)
	agricultural	490 (44.2)	529 (49.1)	8 (21.6)	20 (60.6)	
	non-agricultural	618 (55.8)	548 (50.9)	29 (78.4)	13 (39.4)	
Floating scope						22.240 (0.004)
	trans-provincial	932 (84.1)	863 (80.1)	34 (91.9)	31 (93.9)	
	trans-city	120 (10.8)	162 (15.0)	2 (5.4)	1 (3.0)	
	trans-county	56 (5.1)	52 (4.8)	1 (2.7)	1 (3.0)	
Floating reasons						28.409 (0.005)
	work and do business	171 (16.9)	205 (20.2)	4 (12.1)	14 (48.3)	
	take care of children/grandchildren or float with family members	504 (49.7)	495 (48.7)	19 (57.6)	12 (41.4)	
	treat diseases	9 (0.9)	6 (0.6)	0 (0.0)	0 (0.0)	
	live a retired life	330 (32.5)	310 (30.5)	10 (30.3)	3 (10.3)	
Main economic source						50.055 (< 0.001)
	labor income	130 (11.7)	174 (16.2)	5 (13.5)	15 (45.5)	
	savings/rent	18 (1.6)	15 (1.4)	1 (2.7)	0 (0.0)	
	pension	633 (57.1)	541 (50.2)	25 (67.6)	10 (30.3)	
	subsistence allowance	15 (1.4)	17 (1.6)	1 (2.7)	1 (3.0)	
	family members	262 (23.6)	272 (25.3)	4 (10.8)	5 (15.2)	
Number of friends there						29.747 (0.003)
	none	112 (10.2)	147 (13.8)	8 (21.6)	6 (18.8)	
	1 ~ 5	449 (40.8)	455 (42.8)	12 (32.4)	10 (31.3)	
	6 ~ 10	287 (26.1)	283 (26.6)	11 (29.7)	13 (40.6)	
	> 10	253 (23.0)	179 (16.8)	6 (16.2)	3 (9.4)	
Insurance type						97.155 (< 0.001)
	new rural cooperation	347 (31.3)	424 (39.4)	6 (16.2)	16 (48.5)	
	cooperation between urban and rural residents	44 (4.0)	33 (3.1)	3 (8.1)	0 (0.0)	
	medical insurance for urban residents	131 (11.8)	94 (8.7)	4 (10.8)	1 (3.0)	
	medical insurance for urban workers	288 (26.0)	310 (28.8)	10 (27.0)	6 (18.2)	
	free medical service	31 (2.8)	22 (2.0)	2 (5.4)	0 (0.0)	
	uninsured	267 (24.1)	194 (18.0)	12 (32.4)	10 (30.3)	
Hypertension or diabetes						14.452 (0.006)
	yes	293 (26.4)	240 (22.3)	13 (35.1)	2 (6.1)	
	no	815 (73.6)	837 (77.7)	24 (64.9)	31 (93.9)	

In the case of minor illness, those with primary school education chose to buy medicine or self-treatment while those with high school or technical secondary school education were more likely to see a doctor. The proportion of non-agricultural floating elderly who chose to see a doctor and take medicine from their hometown was higher than that of agricultural floating elderly, while agricultural floating elderly were more inclined to buy medicine or self-treatment. Furthermore, for the floating elderly who carried out trans-provincial floating, the proportion to see a doctor was higher than those who executed trans-city floating, while the trans-city floating elderly were more likely to buy medicine or self-treatment. In addition, the elderly who migrated to work and do business displayed the lowest proportion of seeing a doctor. They were more inclined to buy medicine or self-treatment, and the proportion of no treatment was higher than other groups. Compared with the floating elderly whose main economic source was labor income, the floating elderly dependent on pension had a higher proportion of seeing a doctor, while those who relied on labor income were more inclined to buy medicine or self-treatment, and the proportion of no treatment was also higher than those who relied on other members or pension.

The floating elderly with more than 10 local friends had the highest probability of seeing a doctor, while those who had no friends or 1–5 friends were more willing to buy medicine or self-treatment. In terms of the medical insurance, the proportion of seeing a doctor was the highest among migrant elderly who participated in urban residents’ medical insurance when they had minor illness. Interestingly, the proportion of uninsured floating elderly visiting doctors was higher than that of those who participated in the new rural cooperation. Furthermore, the proportion of the floating elderly who participated in the new rural cooperative medical system or urban employee medical insurance to buy medicine or self-treatment was higher than that of the uninsured elderly. Compared with the floating elderly who participated in the urban residents’ medical insurance, the proportion of the floating elderly who bought medicine or self-treatment was higher. The proportion of the elderly with chronic diseases choosing to see a doctor was higher than that of the elderly without chronic diseases, while the floating elderly without chronic diseases tended to buy medicine or self-treatment, and the proportion of no treatment was higher than that of those who suffered from chronic diseases.

The different influencing factors of the in-patient service utilization among the floating elderly included major income source and hypertension as well as diabetes. However, upon comparison with the floating elderly who were living only on labor related income, the floating elderly who were dependent on pension and on the family members for the financial support were significantly more likely to use in-patient service, which was observed to be 6.27 and 9.56 times, respectively.

## Discussion

### The floating elderly were observed to be generally in good health but a high prevalence of hypertension or diabetes was observed among them

The results in this study further showed that the self-assessed health status of floating elderly decreased significantly with a concomitant increase in the age. In this survey, a very substantial proportion of the floating elderly reported having a good health status, but objectively, the proportion of chronic diseases such as hypertension and diabetes was high, up to 24.2%. Moreover, about 7% of the floating elderly suffered from diseases that required hospitalization. Therefore, the subjective health self-assessment of the elderly floating population in China is too optimistic, and the quality of life of the elderly floating population needs to be improved. The younger age structure may be one of the reasons for this observation. In this survey, 77.4% of floating elderly analyzed belonged to the low-age elderly group, so the overall self-assessed health status was relatively noted to be positive. Moreover, 95.2% of the elderly were found to move regularly across the provinces or megacities. The large-scale floating trend in general needs a greater requirement for maintenance of proper health status. This may also lead to better overall self-assessed health outcomes, which was found to be consistent with prior reported research that the dynamic behavior of floating itself indicated a preference for better health status [16]. The floating elderly had a higher prevalence of both hypertension and diabetes, and the middle-age and high-age elderly generally experienced a greater risk of these lifestyle related diseases. The prevalence rate of diseases which requiring hospitalization for at least in middle-age and high-age floating elderly within 1 year was significantly higher as compared to that observed in the low-age elderly. The results depicted a natural pattern of gradual physical decline with age and reduced functional fitness. Therefore, it was suggested that primary medical and health institutions should provide targeted health services to improve the overall wellness according to the characteristics of floating elderly belonging to the different age groups. For example, providing preventive health care services for low-age floating elderly can play a key role in early detection and treatment of various chronic diseases. In addition, significantly greater attention should be paid to middle-age and high-age floating elderly and they should be regularly provided with personalized medical and health services such as diagnosis and treatment for chronic diseases and subsequent follow-up visits, so as to improve their self-assessed health and wellness levels.

### The health level of the floating elderly was observed to be different between urban and rural areas and between different regions

The results in this study further indicated that compared with the non-agricultural household registration floating elderly, the self-assessed health status of agricultural household registration floating elderly was noted to be relatively poor. Agricultural household registration floating elderly generally engage in manual labor work regularly, but were also found to lack basic health care awareness. They were also observed to have relatively lesser updated health information and medical resources, and hence their self-assessed health level was basically low. The self-assessed health status of floating elderly located in Beijing and Shanghai was noticed to be relatively good. However, the in-patient service utilization rate of the floating elderly in Shanghai was significantly lower than that in Beijing, but the physical examination coverage rate among the floating elderly in Shanghai and Beijing was significantly lower than that in Dalian and Guiyang. The proportion of floating elderly in Guiyang with hypertension or diabetes was found to be low, and the follow-up service utilization rate of hypertension or diabetes was significantly higher than that in Beijing. These phenomena may be possibly related to the uneven distribution of high quality medical and health resources in urban and rural areas and the potential differences in economic development of these various regions with elderly in better developed provinces displaying a better awareness about their general health. Therefore, proper health policies for the floating elderly should be carefully formulated based on their specific needs.

The key to promoting the relevant public reform of registered residence system is the equalization of public services. This is the value and difficulty of registered residence reform. On a deep level, the barrier between urban and rural areas is still a dilemma, which is fundamentally caused by the unbalanced development. In order to solve this problem step by step, the household registration system based on habitual residence could be implemented to guide the population to settle down in an orderly way. For cities with a small permanent population, the restrictions on settlement can be gradually abolished. For example, people who have worked and lived in this city for 10 or 15 years can settle down with their residence permit or social security certificate even if they don’t have a real estate certificate. The specific time limit and conditions for settlement shall be determined by each city.

In addition, the accumulation and transfer system of household registration is a key innovation. If an employee works in city A and then transfers to city B, he can bring all the “relationship” of city A to city B. The subtext is equal service, including social security, medical insurance, education and so on, and to carry forward with the green light all the way to realize real urbanization. Such a registered residence policy helps to promote population movement in the urban agglomerations, and remove a major obstacle for the formation of the high standard market system, which will make the development of the city more viable. Meanwhile, it is necessary to improve the national unified social insurance public service platform and promote the transfer and continuation of social security. Equally important is to build a medical security information system to form a unified and multi-level data sharing and exchange system, so as to promote cross regional, cross level and cross departmental business collaboration, and provide a hard support for registered residence reform.

### The cultivation of health awareness was found to be of great significance contributing to the improvement of the overall health level among the floating elderly

This study also carefully measured the health awareness and health beliefs among the floating elderly population based on their ability to acquire basic health knowledge and the average exercise time daily. Among the 9 different categories of health knowledge, such as occupational disease prevention and nutritional health, approximately more than 60% of the floating elderly were observed to be exposed to 4 categories or less, and generally had an insufficient access to the health knowledge. The floating elderly with high scores in health knowledge acquisition had a strong awareness related to the preventive health care, and were more likely to actively participate in free physical examinations, which may be beneficial to their overall health. In addition, compared with the floating elderly with high education level, the floating elderly who did not attend the school were found to lack health knowledge, and hence their self-rated health performance was generally poor. It was noted that elderly subjects with a higher education level, displayed a significantly stronger awareness related to health risk prevention, and hence these floating elderly with high education level could potentially maintain a better self-assessed health level. The high proportion of the floating elderly with high educational level who regularly undertook physical examinations may be one of the major possible reasons for the relatively higher rate of diagnosed hypertension or diabetes among them. The self-assessed health status of the floating elderly who generally did exercise every day was found to be relatively good. Interestingly, it was found that the longer the exercise time, the more adequate was the utilization of follow-up services for hypertension or diabetes, which was relatively consistent with the previous studies that people with chronic diseases tended to take the initiative to strengthen exercise, and the self-assessed health status of exercisers was significantly better than those who never did any kind of exercise [17]. Therefore, it may be necessary to significantly strengthen the health education level among the floating elderly, with greater emphasis on making them familiar with the general health knowledge related to both the prevention and treatment of chronic diseases. Thus, through the diversified publicity methods such as banners, posters, radio, newspapers, etc., the floating elderly could be successfully trained and guided to actively participate in health exercises and to maintain a healthy lifestyle, so as to improve their overall health and wellness levels.

### The basic medical insurance coverage for the floating elderly was low, and the medical and health services were found to be severely underutilized

Basic Medical Insurance^1^ can greatly reduce the medical expenses of the floating elderly and increase their utilization rate of medical and health related services to promote overall wellness [18]. According to the China Floating Population Development Report 2016, in 2015, the participation rate of floating population in the medical insurance schemes was approximately 89.3%. However, in this survey, the floating elderly who did not participate in any form of medical insurance constituted up to 21.5%, and the coverage rate of basic medical insurance was thus substantially low. In addition, few floating elderly subjects paid a little attention to the basic medical insurance, which may be due to their lack of knowledge related to the benefits of the basic medical insurance. It can also be attributed to the fact that the basic medical insurance may not provide complete security for the floating elderly population due to the difficulty in obtaining a timely reimbursement for off-site medical treatment, and hence they failed to make proper use of the basic medical insurance schemes [19]. In this study, the participation rate of floating elderly in the free physical examination was noted to be only 29%, and the utilization rate of follow-up services for hypertension or diabetes was approximately 28.6%. Interestingly, compared with the floating elderly who participated in free medical services and medical insurance for urban workers, the uninsured floating elderly displayed a significantly lower screening rates for hypertension or diabetes. Moreover, only half of the floating elderly chose to consult a doctor for minor ailments such as fever, diarrhea, etc. Nearly 30% of the floating elderly failed to avail proper in-patient services when suffering from diseases requiring hospitalization such as gastric cancer, fracture, etc. In order to solve the major problem associated with the insufficient medical and health service utilization by the floating elderly, a vigorous publicity campaign should be conducted to popularize the basic medical insurance system, improve the overall coverage rate of basic medical insurance for the floating elderly subjects, and rapidly speed up the process of enrollment in universal medical insurance. According to the China Report on the Work of the Government 2021, in 2020, China’s total expenditure on aiding the poor to participate in insurance has reached 36.9 billion yuan, and the social security fee has been reduced by 1.7 trillion yuan. China continues to promote health and health system construction as one of the priorities of 2021. First, the per capita financial subsidy for residents’ medical insurance and basic public health services increased by 30 yuan and 5 yuan respectively. And continue to give social security subsidies to flexible employment personnel, and promote the liberalization of registered residence restrictions in the employment area. The increase of financial subsidies plays a certain role in meeting the growing health needs, especially in poverty alleviation in poor areas.

Secondly, it was concluded that it is also necessary to improve and standardize the reimbursement process in out-of-town medical treatment cases, facilitate the rapid exchange and sharing of medical insurance related information in different places, and appropriately increase the reimbursement amount to floating elderly to reduce their medical treatment burden and to minimize the cost of their medical related expenses. On the one hand, the direct settlement of medical insurance across provinces meets the medical needs of the floating population at this stage, on the other hand, it is conducive to the free flow of talents and promotes the balanced development of local economy. Finally, more special attention should be paid to the floating elderly subjects, and services such as free physical examination and chronic disease screening should be extensively publicized among them to significantly improve the awareness and utilization rates of the floating elderly in available basic public health service initiatives and programmes.

### Adequate social support can promote the health of the floating elderly and improve their utilization of medical and health services

Floating may be often accompanied by drastic changes in the living environment and reconstruction of social relationship. The availability of adequate social support in an entirely new environment may have an important impact on the overall health status of the floating elderly [20]. The results of this study showed that the self-assessed health status of the floating elderly with active friends was significantly better than those subjects without any friends. Interestingly, it was found that the higher the number of friends the floating elderly had, the lower the rate of diseases requiring hospitalization was. In addition, the number of friends could also significantly improve the participation rate in free physical examination and the utilization rate of follow-up services for hypertension or diabetes among the floating elderly population, and thus encourage them to seek optimal treatment actively for minor ailments. The elderly in the area were observed to be more familiar with the local health services and health resources. The floating elderly could obtain more important health information by actively communicating with health workers in their locality, and this avail the benefits of available medical and health related services. Therefore, the various grassroots organizations such as streets and neighborhood committees should be further strengthened, and the various forms of social activities should be regularly organized, to enhance the sense of belonging among the floating elderly population, and promote their rapid integration into the local society. In addition, different social organizations should be actively mobilized to participate in publicizing their voluntary activities available related to medical and health services. Thus, it is necessary to raise the overall health awareness among the floating elderly population and guide them to make optimal use of various available medical and health services in their locality, so as to improve their overall health and wellness.

### The floating characteristics significantly affected the health of the floating elderly and their utilization rate of the basic public health services

Among the various floating reasons, self-assessed health status of the floating elderly who might be floating to treat existing diseases or live a retired life was generally found to be poor. The rate of diseases requiring hospitalization was significantly higher than that of elderly population floating for other reasons, and the restrictions in economic freedom could be one of the major possible reasons. The floating elderly who took care of their children/grandchildren, floated with their family members, or lived a retired life were observed to be more conscious of their health and took various initiatives to treat their minor ailments. Meanwhile, higher rates of free physical examination also led to a significantly higher screening rates for both hypertension and diabetes. Moreover, upon consideration of the floating scope, the elderly with long distance floating history displayed a significantly better level of health and a lower rate of diseases requiring hospitalization, but their usage of free physical examination was noted to be quite inadequate. The longer the elderly stayed after the move, the more integrated they were into the local community, the more stable their lives were, and the significantly better their self-assessed health status was.

At the same time, it was observed that they made an optimal utilization of the various medical and health services in the inflow area, which was manifested by a higher participation rate in health examination and a significantly higher screening rate for various chronic diseases. Therefore, based on the different floating characteristics of the floating elderly as outlined in this study, targeted policies and measures should be adopted to solve the various problems that may hinder their proper resettlement. For example, medical subsidies should be adequately provided to the floating elderly who suffered from poverty and have low-income due to illness to sufficiently reduce the treatment cost of medical conditions and promote the conversion of health demand into the proper use of available health services. In addition, a more significant attention should be paid to the elderly subjects with short or frequent floating schedule and encourage them to actively participate in social interaction activities and community service initiatives. Overall, it is of utmost necessary to speed up the integration process of the floating elderly into the local society and train them to actively use various preventive health related services, thereby adequately enhancing their health awareness and ultimately improving their overall health and wellness levels.

## Conclusions

At present, the research on the health status and the utilization of medical services among the elderly in China mainly focuses on the elderly in a broader sense. This study was primarily aimed at the floating elderly group, which is major novel aspect of this research object. At the same time, the classical Anderson model was extended to add the floating characteristics that meet the particularity of the research object. The multi-orientation assessment indicators were used to comprehensively measure the health status and medical service utilization of the floating elderly, which can enhance the theoretical and empirical value of the paper and render the conclusions more convincing. In addition, the data was obtained from China Migrants Dynamic Survey-Special investigation on Floating Elderly, which is quite comprehensive and representative and with high reliability and validity. Finally, this paper compared the health status and medical service utilization of the floating elderly in different megacities to a certain extent, thereby clearly revealing the current situation of urban-rural differences and regional differences in China. To sum up, this paper provides an important reference basis for improving the utilization of medical and health services for the floating elderly in the future, which may be of great significance for improving the quality of life for the floating elderly and further promoting the equalization of public services. It can also form an important part of the “Healthy China” strategy and thus prove a key step in the implementation of healthy aging.

## Data Availability

The datasets generated and/or analysed during the current study are available in the *National Population Health Data Center Population Health Data Archive*, https://www.ncmi.cn/phda/dataDetails.do?id=2e6e1f15-f42f-33f7-bed7-cc6aa2c8e337.

## Footnotes

1 In 2015, the basic medical insurance system in China includes the new rural cooperation, urban and rural residents’ cooperation, urban residents’ medical care, and medical insurance for urban workers. In 2016, China began to integrate the urban residents’ medical insurance and the new rural cooperative medical system to establish a unified new medical insurance system, which is called the basic medical insurance for urban and rural residents. At present, most regions have realized the combination of urban residents’ medical insurance and the new rural cooperative medical system. Therefore, at present, there are two kinds of basic medical insurance in China, which refer to the basic medical insurance for urban workers and the basic medical insurance for urban and rural residents.
